# Supplementation With Spirulina Reduces Infarct Size and Ameliorates Cardiac Function in a Pig Model of STEMI

**DOI:** 10.3389/fphar.2022.891801

**Published:** 2022-05-03

**Authors:** Gemma Vilahur, Pablo Sutelman, Soumaya Ben-Aicha, Guiomar Mendieta, Monika Radiké, Leonie Schoch, Laura Casaní, María Borrell-Pagés, Teresa Padro, Lina Badimon

**Affiliations:** ^1^ Cardiovascular Program-ICCC, Research Institute Hospital de la Santa Creu i Sant Pau, IIB-Sant Pau, Barcelona, Spain; ^2^ CiberCV, Institute Carlos III, Madrid, Spain; ^3^ Department of Cardiology, Clinic Hospital, Barcelona, Spain; ^4^ Autonomous University of Barcelona, Barcelona, Spain

**Keywords:** spirulina, STEMI, cardioprotection, antioxidation, anti-inflammatory, anti-apoptotic, weight management

## Abstract

**Background and Aims:** Myocardial infarction (MI) is the clinical manifestation of atherosclerotic coronary artery disease. Spirulina is an algae known to ameliorate cardiometabolic disorders and with proven anti-inflammatory and anti-oxidant effects. We investigated, in a highly translatable animal model, whether oral supplementation with spirulina protects against the deleterious effects triggered by ST-elevation MI (STEMI).

**Methods:** Pigs were fed a regular diet supplemented with spirulina (1 g/animal/bid) or placebo-control for 10 days. Thereafter, animals were subjected to 1.5 h percutaneous balloon-induced coronary occlusion (STEMI) followed by 2.5 h reperfusion and then sacrificed. We assessed infarct size and cardiac function. Blood samples and infarcted and remote myocardial tissue were obtained.

**Results:** Spirulina supplementation reduced infarct size by 64%, increased myocardial salvage by 18%, and improved cardiac function by 30% vs. controls (*p* < 0.05). These benefits were associated with attenuation in DNA-oxidative damage and apoptotic markers and increased iNOS in the infarcted myocardium, higher AMPK activation in the remote myocardium, and lower myocardial MCP-1 expression. Systemically, spirulina attenuated Cox-2 expression in STEMI-activated peripheral blood mononuclear cells and enhanced TNF-α release acutely post-STEMI. Additionally, spirulina decreased weight gain progression over time (*p* < 0.05) without changes in lipids, glucose, liver or kidney parameters.

**Conclusion:** A 10-day supplementation with spirulina exerts cardioprotection in a preclinical setting of STEMI by limiting cardiac damage and improving ventricular contractility through anti-oxidative, anti-inflammatory, and anti-apoptotic mechanisms.

## Introduction

Despite significant improvement in the treatment of atherosclerotic cardiovascular disease (ASCVD), cardiovascular mortality remains the leading cause of death in developed countries (Badimon et al., 2017). Rupture of coronary atherosclerotic plaques and subsequent thrombus formation leads to coronary vessel occlusion and ensuing myocardial infarction (MI). Following an acute ST-segment elevation myocardial infarction (STEMI), the size of the resultant infarction is the major determinant of post-infarct left ventricular dysfunction and the subsequent development of heart failure (Heusch and Gersh, 2017), a condition which exerts a considerable global burden on healthcare and economic resources (Moran et al., 2014). New treatment strategies are needed in order to limit myocardial injury and improve clinical outcomes in patients presenting with acute STEMI.

Nutraceuticals are a group of naturally produced bioactive compounds that have proven health benefits besides their nutritive properties (Badimon et al., 2010; Vilahur et al., 2018; Carrizzo et al., 2020). In this specific cluster, microalgae have stood out as photosynthetic microorganisms capable of generating biofunctional molecules with several cytoprotective activities. Particularly, spirulina, a filamentous cyanobacterium which accounts for up to 30% of the overall microalgal biomass produced worldwide (Costa et al., 2019). Spirulina is primarily comprised of proteins and essential aminoacids providing high nutritional value, but additionally contains phenolic phytochemicals including C-phycocyanin, vitamins, polyunsaturated fatty acids, and an elevated concentration of beta-carotene, delivering substantial antioxidative, anti-inflammatory, and anti-atherosclerotic properties beyond its beneficial effects in metabolic-associated cardiovascular disorders (Finamore et al., 2017; DiNicolantonio et al., 2020). A systematic review including 18 randomized controlled trials has reported that spirulina supplementation is safe and displays positive effects in multiple metabolic syndrome components (Yousefi et al., 2019). As such, spirulina has shown to exert a noteworthy weight management capacity eliciting a reduction in both waist circumference and body mass index in several clinical trials (DiNicolantonio et al., 2020; Grosshagauer et al., 2020). Besides, several studies with spirulina have shown an improvement in insulin sensitivity and glucose uptake mediated by C-phycocyanin activity, and, in some studies spirulina has displayed hypolipidemic properties (Park et al., 2008; Mazokopakis et al., 2014b; Serban et al., 2016). On the other hand, cell culture approaches have demonstrated the ability of spirulina to exhibit anti-atherosclerotic effects by preventing monocyte migration through direct inhibition of P- and E- selectin adhesion molecules (Vo and Kim, 2013; Ren et al., 2018), and to effectively inhibit doxorubicin-induced cardiac damage (Khan et al., 2005; Khan et al., 2006a). However, whether oral supplementation with spirulina is able to limit infarct size and ameliorate cardiac dysfunction in the setting of myocardial infarction remains to be addressed. With this in mind, we sought to examine the potential cardioprotective effects associated with oral daily supplementation with spirulina in a highly translatable preclinical animal model of STEMI.

## Materials and Methods

### Animal Procedures

Animal management was performed following European Directive 2010/63/EU and in accordance with the ARRIVE 2.0 guidelines (Percie du Sert et al., 2020) and complies with the ′3Rs (Animal Ethics Infolink, 2019). Experimental protocols were approved by the Institutional Animal Care and Use Committees.

### Study Design

The experimental design involved crossbred commercial female swine (*n* = 12; Landrace × Largewhite) fed a regular pig chow diet randomly supplemented with spirulina (1 g/animal/bid; *n* = 6) or placebo-control (*n* = 6) for 10 days. Animals were fed a normocholesterolemic diet dosed at 3.5% of pig’s body weight adjusted on a daily basis. The regular chow included: gross protein 15.1%; gross fat 3.40%; gross fiber 4.00%; gross ash 5.50%; calcium 0.81%; phosphorus 0.57%; sodium 0.15%; lysine 0.89%; methionine 0.24%. All pigs were monitored to ensure intake of their chow and exclude any potential bias due to a decrease in food intake. Spirulina capsules were composed of 100% spirulina platensis (Pranarom, ACL 7846467). Placebo tablets were comprised of standard commercially available gelatin capsules. At the end of the treatment period and 2 h after the last dosage, pigs were subjected to a 1.5 h experimental STEMI induction followed by 2.5 h phase of complete reperfusion (i.e., ischemia/reperfusion). Thereafter pigs were sacrificed and cardiac tissue samples collected. The dosage of spirulina administered in this study was selected according to the use in both pigs (Yordanova et al., 2015) and clinical trials (Huang et al., 2018).

### Closed-Chest Animal Model of ST-Segment Elevation Myocardial Infarction

STEMI was experimentally induced by fluoroscopy-guided percutaneous coronary intervention as previously described (Vilahur et al., 2009; Mendieta et al., 2019a; Mendieta et al., 2020) in a preclinical animal model with human resemblance. Briefly, prior to the procedure, pigs were anesthetized with a combination of intramuscular tiletamine + zolazepam (7 mg/kg) + medetomidine (0.07 mg/kg). Animals received a continuous and stable flow of oxygen (inspired fraction of 0.5%) during all the intervention with permanent control of arterial saturation. Anesthesia was maintained with inhalatory isofluorane (2%) throughout the entire procedure (both in the course of myocardial infarction induction as well as in the reperfusion period). Continuous electrocardiographic monitoring with hemodynamic parameters was performed. Coronary occlusion of the mid left anterior descending artery posterior to the emergence of the first diagonal branch was induced by balloon inflation, verifying a Thrombolysis In Myocardial Infarction (TIMI) 0 flow downwards. A period of 1.5 h was sustained with complete coronary obstruction and subsequently reperfusion was achieved by balloon deflation confirming TIMI 3 flow restoration.

### Cardiac Function Evaluation

A Phillips iE33 echocardiography system equipped with a S5-1 Sector Array transducer was employed for the attainment of transthoracic echocardiography in every animal prior to STEMI induction, post-STEMI, and after 2.5 h of reperfusion as previously performed (Vilahur et al., 2014b). Left ventricle ejection fraction (LVEF) was assessed as a surrogate for LV global systolic function. All measurements were acquired by an independent operator blinded to treatment group.

### Cardiac Sample Collection and Myocardial Damage Assessment

After the 2.5 h reperfusion period animals were injected with Evan’s Blue dye to properly define the area-at-risk (AAR) in order to ensure comparable degree of jeopardized myocardium (i.e., ischemic heart). Subsequently, pigs were sacrificed during anesthesia with an intravenous administration of 10 ml KCl 2M. Hearts were carefully excised and segmented in six transverse divisions (1 cm width) in order to alternatively collect slices for 2,3,5-triphenyltetrazolium (TTC) staining (infarct size assessment) or molecular/immunohistochemical analyses. In this latter regard, myocardial samples from both infarcted and remote regions were obtained, frozen, pulverized in liquid nitrogen and minced in Tripure^®^ or lysis buffer for RNA and protein isolation, respectively. The AAR and TTC staining were appraised by an independent blinded observer through planimetry assessment employing ImageJ^®^ software analysis (NIH). Infarct size was expressed as % AAR.

### Myocardial Oxidative Damage

Paraffin-embedded myocardial tissue from the infarcted region was cut and placed on poly-L-lysine-coated slides and deparaffinized. Antigen removal was required before staining for 8-hydroxyguanosine (8OH-G, mouse monoclonal antibody; Abcam ab48508), a sensitive biomarker of intracellular DNA damage induced by oxidative stress. Four images per animal were captured by Nikon Eclipse 80i microscope and digitized by Retiga 1300i Fast camera, and staining of the myocardial tissue was quantified and expressed as % stained area.

### Molecular Footprint of the Infarcted Heart

We assessed in both the infarcted and remote myocardium protein expression levels of: 1) inducible nitric oxide synthase (iNOS); 2) monocyte chemoattractant protein-1 (MCP-1), an inflammatory chemokine; 3) adenosine monophosphate activated protein kinase (AMPK) phosphorylation, a key marker of cardiac metabolism and autophagy; and 4) total and truncated caspase-3, a marker for apoptotic cell death execution. Besides, we also investigated caspase-3 transcript levels by real-time PCR-7000 Sequence Detection System of ABIPRISM (Applied Biosystems) by an assay-on demand. Protein expression was normalized to ß-actin and presented in arbitrary density units (AU) whereas for gene expression analyses, the threshold cycle (Ct) values were determined and normalized to the housekeeping 18SrRNA.

### Systemic Inflammatory Response: Peripheral Blood Mononuclear Cells and Circulating Cytokine Levels

We assessed the impact of spirulina supplementation on peripheral blood mononuclear cells (PBMCs) activation and cytokine release. For this purpose, 20 ml of blood were collected into EDTA tubes at the three tested time points (prior-STEMI, post-STEMI, and 2.5 h post-reperfusion) from all animals. Blood samples were immediately subjected to Ficoll-paque Plus (Amersham Biosciences, Piscataway, NJ, United States) density gradient centrifugations to isolate PBMCs. PBMC were processed for protein analyses of cyclooxygenase 2 (Cox-2) which was normalized to Ponceau staining. We also analyzed tumor necrosis factor-α (TNF-α) and interleukin-1β (IL-1β) levels in plasma using commercially available enzyme-linked immunosorbent assay kits (porcine TNF-α and IL-1β from Quantikine Porcine Kits from R&D Systems). According to the manufacturers, the minimum detection limits for TNF-α and IL-1β were 23.4 and 39.1 pg/ml^−1^, respectively.

### Biochemical Plasma Analysis

Blood samples were obtained at baseline, day 10, prior-STEMI, acutely post-STEMI (5 min following coronary occlusion) and after 2.5 h of reperfusion (sacrifice) for haematological (System 9000, Serono-Baker Diagnostics) and biochemical analysis (glucose and lipid levels, and kidney and liver function parameters; Clima MC-15 RAL Biotechnologies).

### Weight Control

Animals were subjected to weight assessment at baseline and after completion of the 10-day experimental period.

### Statistical Analysis

Continuous variables were expressed as mean ± standard deviation (SD). After testing for normal distribution (Shapiro-Wilk test), repeated ANOVA measures and paired t-test as appropriate were used to analyze variables within each group, whereas unpaired t-test was used for all single time-point measurements. A cut-off value of *p* < 0.05 was used to consider statistical significance. Statistical analyses were performed with the GraphPad Prism software package.

## Results

### Spirulina Supplementation Limits Weight Gain

**FIGURE 1:**
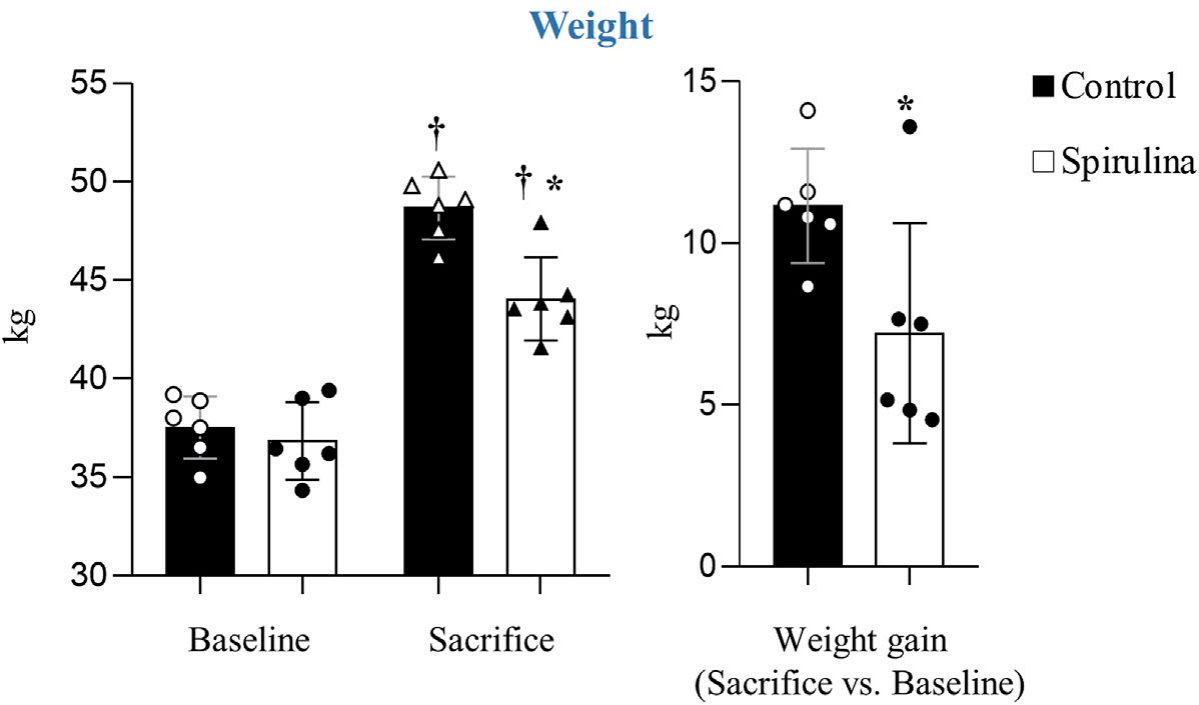
Effects of Spirulina supplementation on weight.∗*p* < 0.05 vs. control; ^†^ *p* < 0.05 vs. baseline. Data expressed as mean ± SD.

Weight gain was significantly lower in animals receiving spirulina supplementation as compared to controls (19% vs. 29%, respectively; *p* = 0.01; Figure 1).

### Spirulina Supplementation Reduces Myocardial Damage and Improves Cardiac Function Acutely Post-ST-Segment Elevation Myocardial Infarction

**FIGURE 2:**
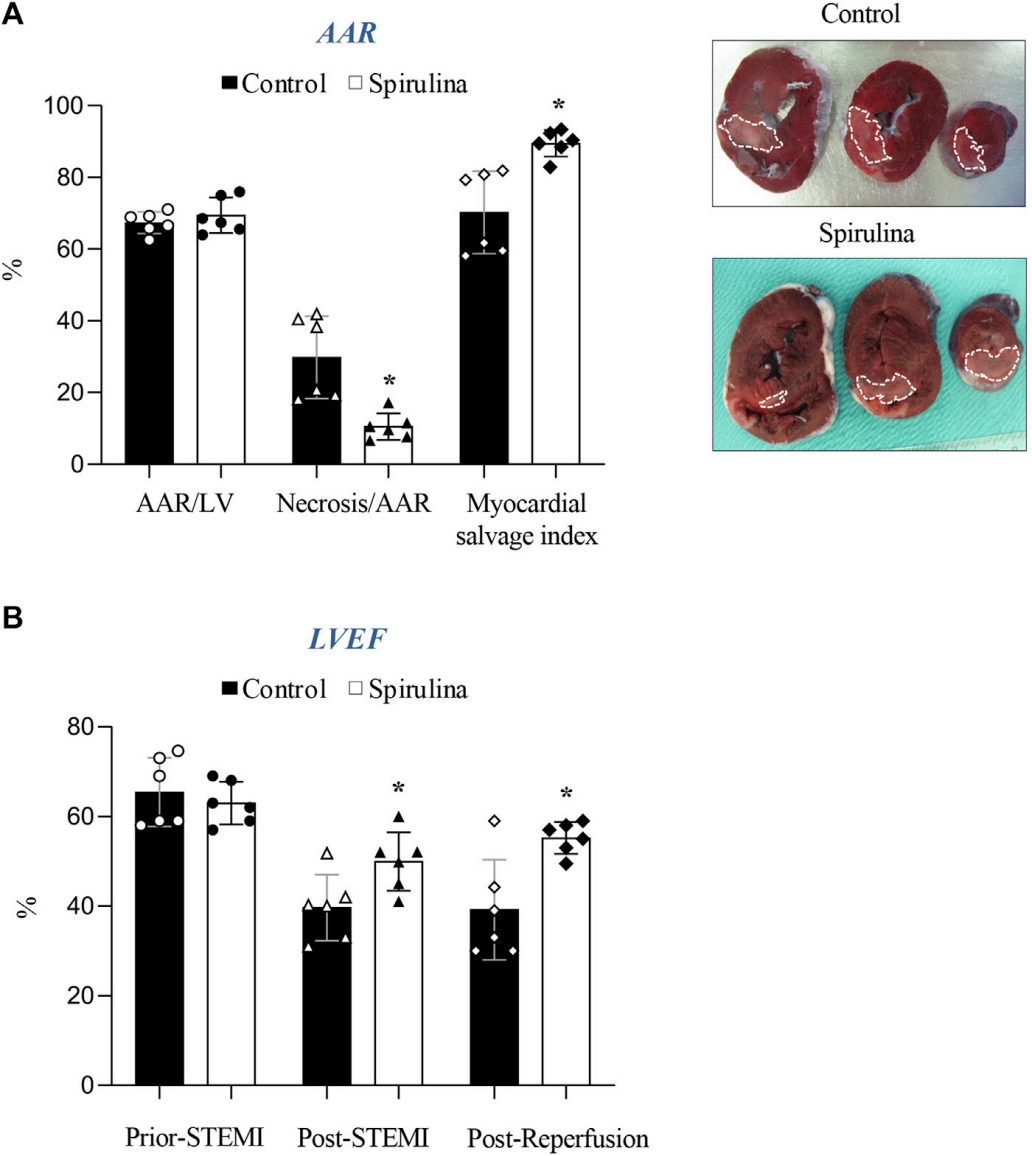
Effect of Spirulina supplementation on cardiac damage and function. **(A)** Effect of Spirulina on infarct size. **(B)** Effect of Spirulina on cardiac function assessed by echocardiography. AAR, area at risk; LV, left ventricle; LVEF, left ventricular ejection fraction; STEMI, ST-elevation myocardial infarction.∗*p* < 0.05 vs. control. Data expressed as mean ± SD.

Both placebo-control and spirulina-treated animals displayed a similar extent of jeopardized myocardium (AAR/LV) (67.7% ± 3.5% vs. 69.4% ± 4.9%, respectively; *p* = 0.5) indicating a comparable severity of balloon-induced myocardial ischemic damage (Figure 2A).

A 10-day supplementation regime with spirulina markedly limited infarct size by 64% as compared to placebo-control animals (29.8% ± 11.5% vs. 10.5% ± 3.7% AAR, respectively; *p* = 0.007). Accordingly, myocardial salvage was 19% greater in the spirulina group as compared to placebo-control animals (*p* < 0.05).

The overall reduction in myocardial damage was associated with a 38% improvement in LVEF vs. controls, an effect already detected post-STEMI that persisted up to 2.5 h post-reperfusion (*p* < 0.05; Figure 2B).

### Spirulina Supplementation Attenuates Myocardial DNA-Oxidative Damage

**FIGURE 3:**
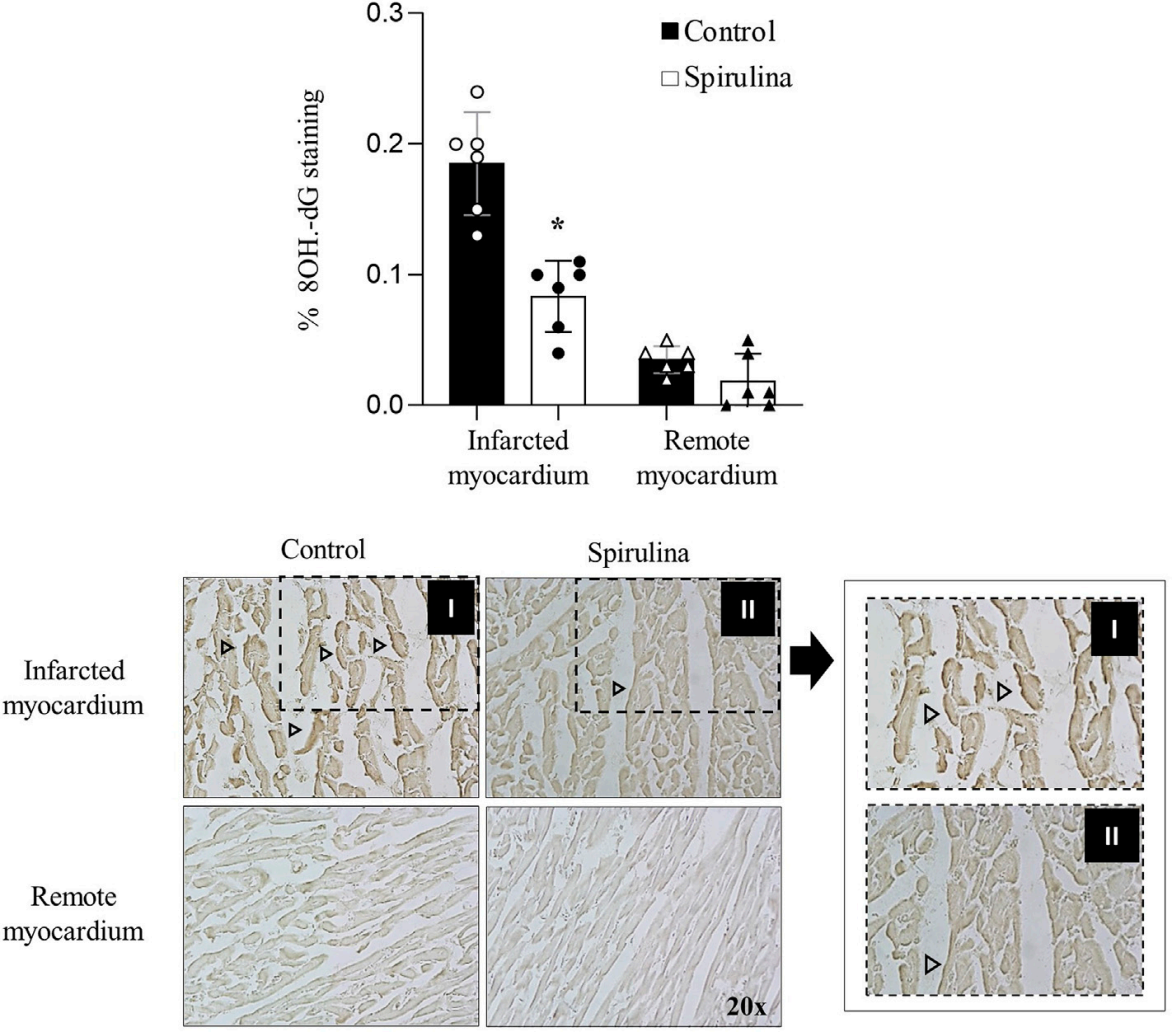
Antioxidant effects of Spirulina on the infarcted myocardium. Effects of Spirulina on DNA oxidative damage assessed by 8 hydroxydeoxyguanosine (8OH-dG) staining.∗*p* < 0.05 vs. control. Data expressed as mean ± SD. Arrow heads depict 8-OH-dG statining.

Spirulina supplemented-animals displayed a marked 58% reduction in oxidative DNA damage in the infarcted myocardium as compared to placebo-control animals (*p* < 0.05; seen in dark-brown in Figure 3). 8OH-dG staining was barely detected in the remote myocardium of all pigs.

### Effects of Spirulina on Myocardial Molecular Footprint

**FIGURE 4:**
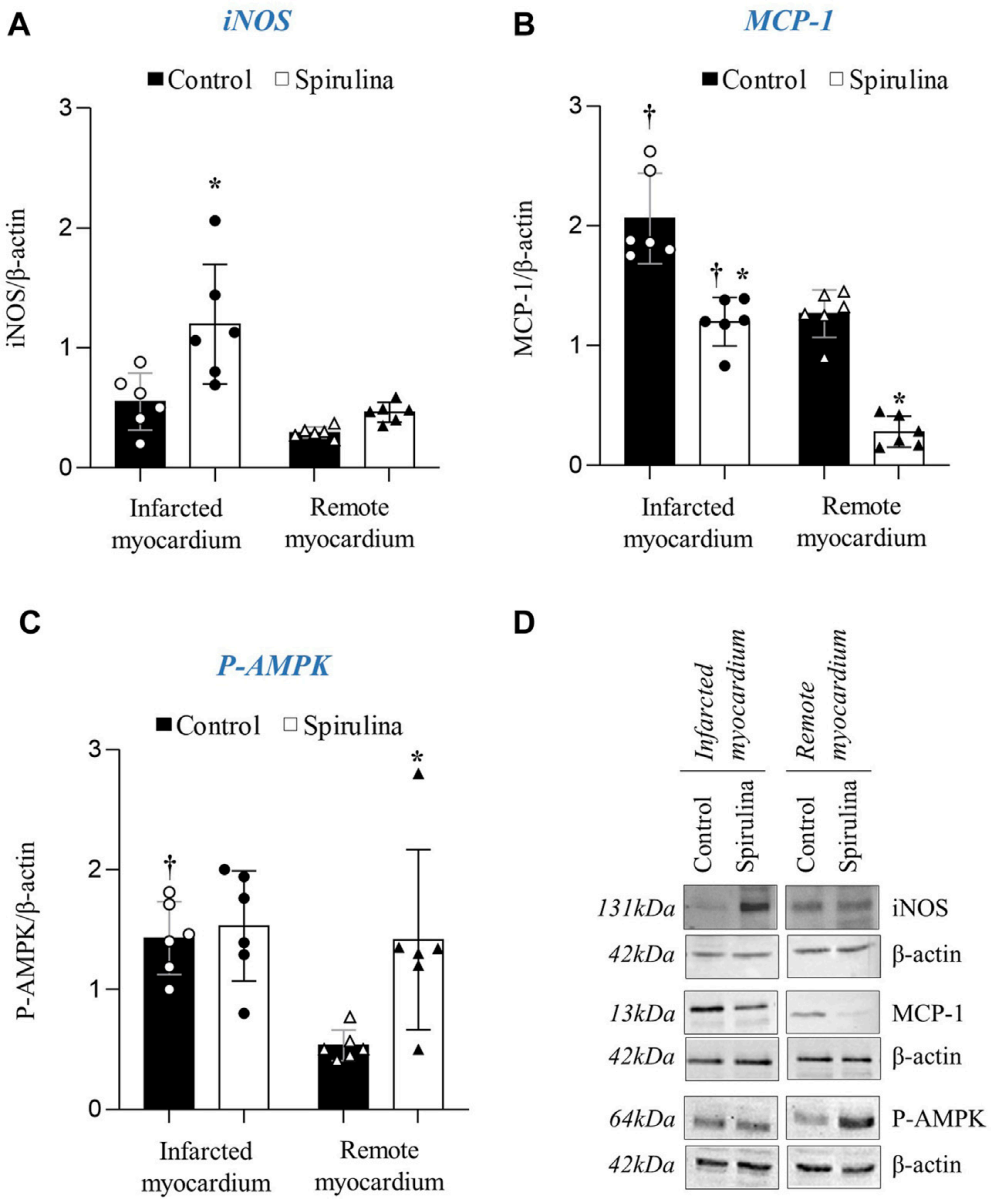
Effect of Spirulina on myocardial molecular footprint. Myocardial protein expression of inducible nitric oxide synthase iNOS; **(A)** monocyte chemoattractan protein-1 MCP-1; **(B)** and Phosphorylated AMP protein kinase P-AMPK; **(C)** in the infarcted and remote myocardium. **(D)** Representative Western Blot images.∗*p* < 0.05 vs. control; ^†^ *p* < 0.05 vs. remote myocardium. Data expressed as mean ± SD.

We assessed protein levels of iNOS, MCP-1, and P-AMPK. As observed in Figures 4A, D, spirulina supplementation led to a significantly higher iNOS protein level in the infarcted region (*p* < 0.05 vs. control animals) whereas exerted no impact on the remote myocardial tissue. Regarding MCP-1, spirulina supplemented-animals showed a significant reduction in both the infarcted and remote myocardium as compared to the control arm (*p* < 0.05; Figures 4B–D). P-AMPK showed higher levels in the infarcted myocardium of control pigs as compared to the remote myocardial region (*p* < 0.05) whereas levels were higher in the entire left ventricle of spirulina supplemented pigs (*p* < 0.05 vs. remote cardiac tissue from control pigs; Figures 4C,D).

### Spirulina Supplementation Reduces Apoptosis Execution in the Infarcted Cardiac Region

**FIGURE 5:**
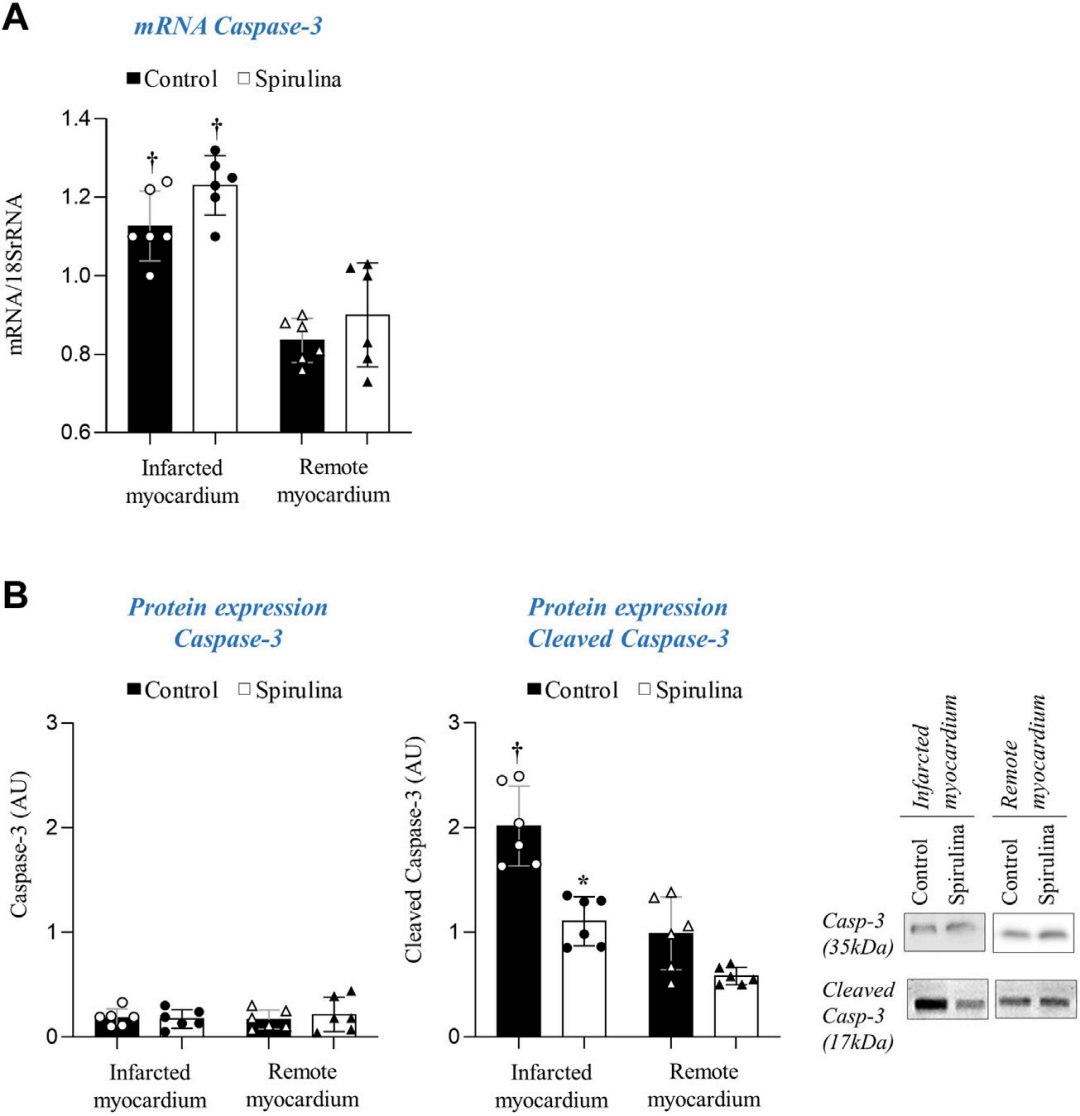
Effect of Spirulina on myocardial apoptosis execution. **(A)** Caspase-3 transcript levels. **(B)** Protein expression of total and cleaved caspase-3.∗*p* < 0.05 vs. Spirulina; ^†^ *p* < 0.05 vs. remote myocardium. Data expressed as mean ± SD.

Caspase-3 mRNA was found to be upregulated in the infarcted tissue of all animals as compared to remote cardiac regions; yet, no differences were detected in caspase-3 protein expression (Figures 5A,B). In contrast, spirulina supplementation was associated with a significant lower levels of truncated caspase-3 in the infarcted cardiac region as compared to controls (*p* < 0.05; Figure 5C). No changes were observed in active caspase-3 in the remote myocardium of both animal groups.

### Spirulina Supplementation Exerts Systemic Anti-Inflammatory Effects

**FIGURE 6:**
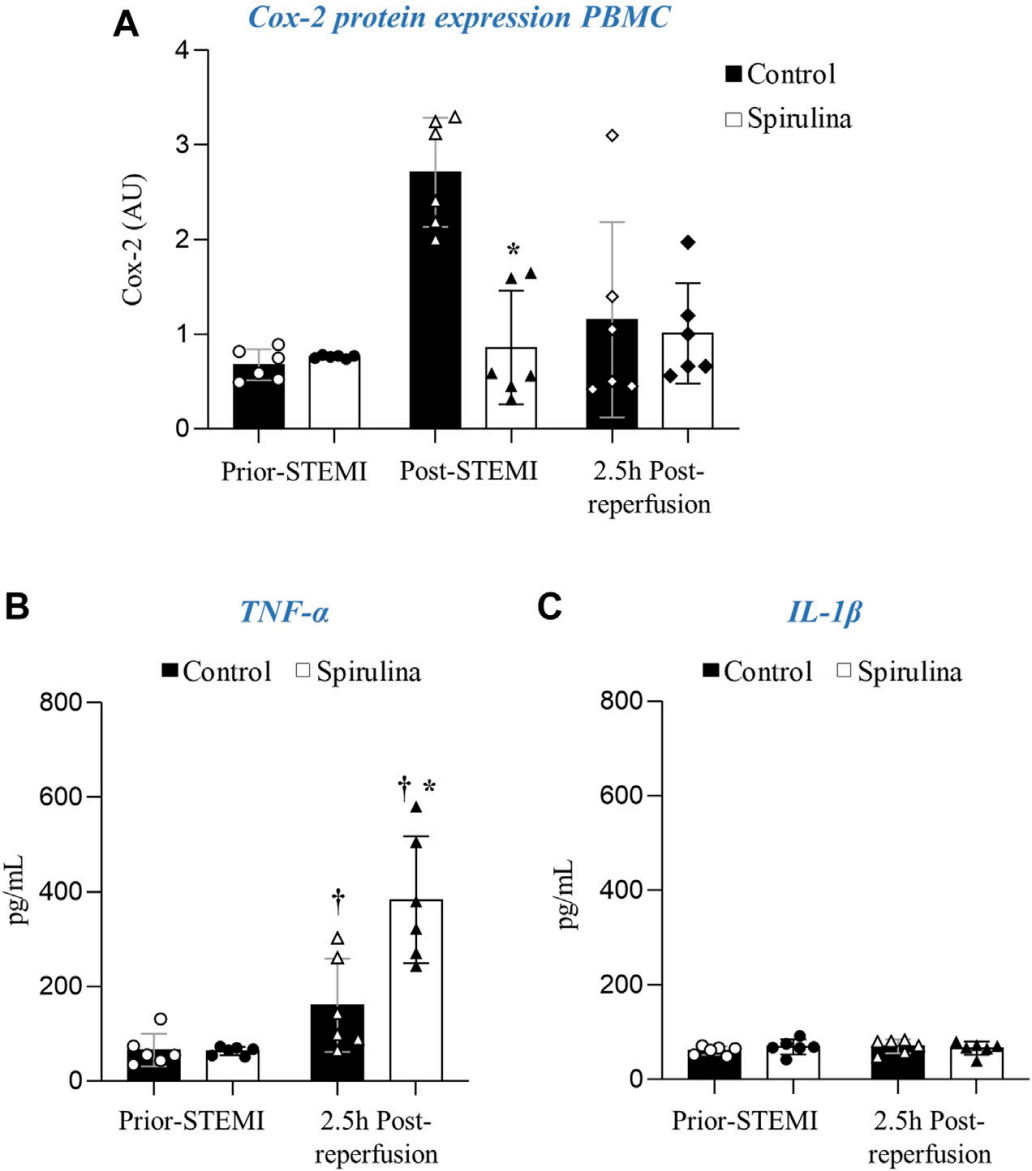
Effect of espirulina on the systemic inflammatory response. **(A)** Cox-2 protein expression in peripheral blood mononuclear cells (PBMC). Circulating levels of **(B)** TNF-α and **(C)** IL-1β.∗*p* < 0.05 vs. control; ^†^ *p* < 0.05 vs. prior-MI. Data expressed as mean ± SD.

Control animals showed an induction of Cox-2 protein expression in PBMCs acutely post-STEMI that was not found in spirulina supplemented animals (Figure 6A). All animals showed higher circulating levels of TNF-α 2.5 h post-reperfusion as compared to baseline, although these levels were significantly higher in the spirulina supplemented subgroup as compared to controls (*p* < 0.05). No changes were detected in IL-1β circulating levels in both groups at all tested time points (Figure 6B).

### Spirulina Effects on the Metabolic Profile, and Hematological and Biochemical Parameters

Liver, kidney, lipid and glucose levels and hematological parameters were all within physiological ranges throughout the entire study (Table 1).

**TABLE 1:** Haematological parameters, **(A)** liver and kidney function markers, **(B)** plasma glucose levels and lipid profile **(C)** at baseline, 10 days, prior-STEMI (ST-elevation myocardial infarction), 5 min post-STEMI and at 2.5 h post-reperfusion (sacrifice).

	Time	Control	Spirulina	*p* value
A. Haematological parameters				
Hematocrit (vol %)	Baseline	27.0 ± 0.9	25.6 ± 0.5	0.20
	10 days	25.8 ± 1.2	24.8 ± 1.4	0.60
	Prior-STEMI	25.7 ± 0.1	23.8 ± 0.9	0.09
	Post-STEMI	26.9 ± 1.6	22.2 ± 0.9	0.03
	Sacrifice	25.8 ± 1.2	24.8 ± 1.4	0.60
Hemoglobin (g/dl)	Baseline	9.7 ± 0.6	9.3 ± 0.4	0.63
	10 days	9.7 ± 0.54	9.1 ± 0.5	0.45
	Prior-STEMI	10.8 ± 0.1	8.9 ± 0.6	0.03
	Post-STEMI	9.5 ± 0.5	8.3 ± 0,4	0.08
	Sacrifice	9.7 ± 0.5	9.1 ± 0.5	0.45
White blood cells (10^9^/L)	Baseline	15.0 ± 1.9	14.4 ± 1.3	0.81
	10 days	16.5 ± 2.4	11.3 ± 1.5	0.10
	Prior-STEMI	29.2 ± 7.2	14.3 ± 1.8	0.27
	Post-STEMI	14.8 ± 2.3	11.9 ± 2.1	0.36
	Sacrifice	16.5 ± 2.4	11.3 ± 1.5	0.10
Platelet count (10^9^/L)	Baseline	245.5 ± 27.5	354.2 ± 28.2	0.02
	10 days	232.1 ± 28.6	324.8 ± 31.6	0.06
	Prior-STEMI	291.5 ± 79.5	362.7 ± 33.6	0.53
	Post-STEMI	236.8 ± 29.8	317.1 ± 26.7	0.07
	Sacrifice	232.1 ± 28.6	324.8 ± 31.6	0.06
B. Kidney and liver function markers				
Blood urea nitrogen (mg/dl)	Baseline	11.0 ± 2.1	17.1 ± 4.0	0.22
	10 days	15.7 ± 7.8	15.7 ± 4.7	0.99
	Prior-STEMI	24.8 ± 2.8	17,2 ± 2,3	0.14
	Post-STEMI	15.0 ± 3.1	17.6 ± 3.1	0.56
	Sacrifice	15.7 ± 7.8	15.7 ± 4.7	0.99
Creatinine (mg/dl)	Baseline	1.0 ± 0.1	1.2 ± 0.1	0.12
	10 days	1.2 ± 0.1	1.3 ± 0.1	0.44
	Prior-STEMI	1.0 ± 0.2	1.3 ± 0.1	0.53
	Post-STEMI	1.2 ± 0.1	1.5 ± 0.2	0.15
	Sacrifice	1.2 ± 0.1	1.3 ± 0.1	0.44
Alanine aminotransferase (U/L)	Baseline	39.3 ± 11.8	35.9 ± 2.6	0.78
	10 days	37.2 ± 0.3	35.2 ± 17.0	0.91
	Prior-STEMI	45.9 ± 2.2	50.0 ± 16.9	0.81
	Post-STEMI	27.7 ± 4.4	26.6 ± 4.0	0.85
	Sacrifice	37.2 ± 0.3	35.2 ± 17.0	0.91
C. Plasma glucose levels and lipid profile				
Glucose (mg/dl)	Baseline	95.6 ± 11.9	118.5 ± 7.2	0.13
	10 days	95.6 ± 11.9	112.8 ± 23.2	0.56
	Prior-STEMI	76.2 ± 10.1	108.7 ± 8.6	0.09
	Post-STEMI	129.8 ± 14.5	140.2 ± 12.1	0.59
	Sacrifice	95.6 ± 11.9	112.8 ± 23.1	0.56
Total cholesterol (mg/dl)	Baseline	97.5 ± 3.9	82.4 ± 6.9	0.10
	10 days	95.6 ± 12.9	76.7 ± 5.7	0.27
	Prior-STEMI	78.5 ± 17.5	84.5 ± 6.2	0.78
	Post-STEMI	90.6 ± 7.6	83.1 ± 5.4	0.44
	Sacrifice	95.6 ± 12.9	76.7 ± 5.7	0.27
Triglycerides (mg/dl)	Baseline	25.8 ± 3.9	35.0 ± 7.3	0.37
	10 days	28.3 ± 4.3	32.5 ± 2.5	0.49
	Prior-STEMI	22.5 ± 0.5	23.6 ± 3.1	0.83
	Post-STEMI	26.8 ±2.4	29.6 ± 3.6	0.67
	Sacrifice	28.3 ± 4.3	32.5 ± 2.5	0.49
High-density lipoprotein cholesterol (mg/dl)	Baseline	35.9 ± 4.2	47.9 ± 13.2	0.43
	10 days	57.8 ± 8.0	64.5 ± 4.4	0.51
	Prior-STEMI	21.6 ± 4.7	42.9 ± 7.4	0.06
	Post-STEMI	31.0 ± 6.8	47.4 ± 7.5	0.13
	Sacrifice	57.8 ± 8.0	64.5 ± 4.4	0.51
Low-density lipoprotein colesterol (mg/dl)	Baseline	85.2 ± 16.2	56.2 ± 16.5	0.25
	10 days	96.2 ± 12.1	51.0 ± 14.22	0.06
	Prior-STEMI	52.3 ± 12.8	58.3 ± 8.7	0.73
	Post-STEMI	78.8 ± 17.0	64.1 ± 10.2	0.47
	Sacrifice	96.2 ± 12.1	51.0 ± 14.2	0.06

Data are expressed as mean ± SD. *p* value denotes differences between control pigs and spirulina-treated animals. All values are within physiological ranges based on our in-house data for young healhy pigs.

## Discussion

Atherosclerotic plaque rupture is the most common cause of MI (Vilahur et al., 2014a). MI-size largely determines patient’s outcome. Hence, efforts have focused on the identification of strategies able to limit myocardial damage. In the following study we demonstrate for the first time that a 10-day oral supplementation with spirulina exerts cardioprotection in a preclinical setting of STEMI by enhancing myocardial salvage and improving ventricular contractility through antioxidative, anti-inflammatory, and anti-apoptotic mechanisms.

Spirulina is a blue-green algae increasingly employed as a nutritional supplement because of the health outcomes associated to its regular intake, particularly regarding metabolic syndrome components (Khan et al., 2006b; Stone et al., 2016). Herein, we further prove that a 10-day supplementation with spirulina significantly limits coronary artery occlusion-related cardiac damage (i.e., infarct size). The size of infarction is strongly related to mortality and heart failure development in STEMI patients (Heusch et al., 2014). Despite efforts have focused on testing therapies to promote myocardial salvage, currently there is no therapy routinely used in the clinical setting (Heusch and Gersh, 2020).

So far, the *in vivo* effects of spirulina on the myocardium have been only investigated in a mice model of doxorubicin-induced cardiotoxicity where oral spirulina supplementation for 7 weeks elicited a decrease in lipid peroxidation, normalized antioxidant enzymes, and limited doxorubicin-induced myocardial ultrastructural changes leading to an overall significant reduction in mortality (Khan et al., 2005). In our study we further expand spirulina-related cardioprotective properties to the setting of coronary artery total occlusion and elucidate the mechanisms behind its action. As such, we have evidenced that animals receiving spirulina supplementation have significantly higher iNOS protein levels and a diminished myocardial oxidative injury in the infarcted heart and a higher AMPK activation levels in the entire myocardial tissue. Activation of iNOS/NO signaling in the setting of MI has shown to exert both protective and detrimental effects (Yu et al., 2018). On the one hand, there is overwhelming evidence supporting the ability of NO-derived iNOS to mediate the anti-stunning, vasodilator and anti-infarct effects in preconditioned hearts (i.e., hearts more resistant to sustained ischemia-related damage because of the previous exposure to multiple short episodes of ischemia and reperfusion) (Xi et al., 1999; Bolli, 2001). In fact, iNOS was the first gene identified to mediate late ischemic pre-conditioning (Madonna et al., 2015), and gene therapy with iNOS, either 3 days or up to 2 months prior-MI induction (Li et al., 2006; Li et al., 2011), has shown to exert cardioprotection. Yet, it should also be considered that enhanced iNOS/NO may lead to peroxynitrite formation and subsequently associated oxidative damage. However, in such scenario, the powerful antioxidant properties of spirulina might have blunted iNOS-related oxidative stress thereby switching iNOS from detrimental to protective and consequently enhancing the ability of the heart to withstand STEMI-related injury. Nevertheless, further research is warranted to determine the mechanism by which spirulina acutely induces a higher iNOS expression in the ischemic myocardium in the setting of MI. TNF-α, a cytokine known to acutely protect against infarction and contractile dysfunction, has shown to regulate iNOS (Lecour et al., 2002). Here, we detect a significant and acute increase in TNF-α plasma levels with an enhanced iNOS expression and reduced oxidative damage in the infarcted heart of spirulina supplemented animals. TNF-α has an ambivalent role in MI. Transient increase in TNF-a has shown to protect against ischemia–reperfusion injury by interacting with myocardial TNF receptor type 2 and consequent downstream activation of the cardioprotective SAFE pathway (Lecour et al., 2005) whereas excessive and long-lasting release of TNF-α has shown to exert detrimental effects (Schulz and Heusch, 2009). Moreover, we also evidence higher AMPK activation in the remote myocardium of spirulina supplemented animals indicating that spirulina cardioprotective benefits are not only confined to the infarcted region but to the entire left ventricle. We and others have reported that AMPK signaling, besides being essential to maintain cardiac energy metabolism under ischemic stress, induces autophagy protecting against the progression of post-STEMI adverse cardiac remodeling (Kanamori et al., 2011; Vilahur et al., 2016; Mendieta et al., 2019b). A recent study has demonstrated the ability of spirulina to induce mRNA expression levels of autophagy-associated genes (i.e., AMPK and ULK1) in the liver of growing lambs, supporting our findings in the heart tissue (Liang et al., 2020). We also identify lower levels of the inflammatory marker MCP-1 in the entire myocardium of spirulina supplemented pigs. Furthermore, spirulina anti-inflammatory effects are also evident systemically by a reduced Cox-2 expression in the acute phase after coronary occlusion in PBMCs. Of note, no changes were observed in IL-1β plasma values among all animals at the different tested time points. In this regard, a low intrinsic expression of myocardial NLRP3 inflammasome (involved in processing of pro-IL-1β into its active form) has been detected in the acute phase of infarcted hearts (Jong et al., 2014), and could potentially support the detected low IL-1β levels. Nevertheless, the overall antioxidant and anti-inflammatory effects may explain the reduction in caspase-3 activation observed in the infarcted myocardium of spirulina supplemented pigs which, in turn, has likely contributed to promote myocardial salvage and limit cardiac dysfunction in the setting of STEMI.

It has been claimed that spirulina improves several well-established cardiovascular risk factors providing benefits around weight loss and hyperlipidaemia. A recent systematic review and meta-analysis of five randomized clinical trials assessing spirulina effect on weight loss found that spirulina supplementation significantly decreases total weight, body fat percentage, and waist circumference in obese subjects (Moradi et al., 2019). Of note, there is no clinical evidence to support a potential modulation of spirulina in food intake (Moradi et al., 2019) and, in line with our observations, spirulina has shown to exert no effects in food intake in mice (Zhao et al., 2019). We observe that animals supplemented with spirulina for 10 days displayed a significant decrease in the rate of weight gain progression in comparison to control pigs. Several mechanistic pathways have been proposed to explain spirulina’s ability to improve weight management. As such, spirulina high content of anti-oxidative molecules has shown to partly inhibit lipase activity while modulating appetite and food intake (Fujimoto et al., 2012; Hassan and El-Gharib, 2015). Besides, phenylalanine, one of the essential aminoacids comprised in spirulina structure, has also shown to directly inhibit the brain appetite center (Mazokopakis et al., 2014a). Regarding the hypolipidemic response to spirulina supplementation, this has been observed to differ in clinical trials based on the dosage, duration of treatment, and the presence of comorbidities (Finamore et al., 2017; Hernández-Lepe et al., 2019). A small trial in 30 healthy volunteers with mild hyperlipidaemia demonstrated a notable reduction in total cholesterol after an 8 week supplementation with 4.2 g spirulina, although no changes were reported for HDL-cholesterol or triglyceride levels (Nakaya and Goto, 1988). Another clinical trial in Korean patients demonstrated that a 12 week supplementation with spirulina 8 g/day significantly displayed lipid-lowering activity in non-obese subjects, while in the obese subgroup no substantial change in lipid metabolism was found (Park and Lee, 2016). Moreover, spirulina supplementation for long periods has recently shown to alter the gut microbiota that ultimately affect lipid metabolism and weight (Li et al., 2021). Conversely, a study in obese individuals observed no changes in lipid profile after a 12-week supplementation of spirulina 1 g/day despite a reduction in weight gain. We do not observe any changes in lipid profile or glucose levels after this short-term spirulina administration at doses of 1 gr/animal/bid. Further research is warranted to determine the optimal spirulina dose and timing required to improve glucose metabolism and lipid profile in each specific setting and whether these benefits are associated with changes in the gut microbiome. Importantly, it deserves to be stated that spirulina supplementation for 10 days confirmed its adequate safety profile, and no adverse events were registered, with kidney and liver function remaining unmodified throughout the entire experimental period.

This study was carried out in young and healthy pigs without comorbidities and/or associated medication. We intended to address spirulina’s cardioprotective activity based on its nutraceutical profile and its consumption as a dietary supplement in subjects with no overt cardiovascular diseases exposed to a sudden ischemic event. In fact, spirulina has been proposed as a supplementary nutritional additive in healthy subjects with the aim to improve their overall health status (Karkos et al., 2011). Future studies should investigate the effects of spirulina in normal healthy hearts as well as in clinical scenarios combining metabolic comorbidities and ischemic heart disease.

**FIGURE 7:**
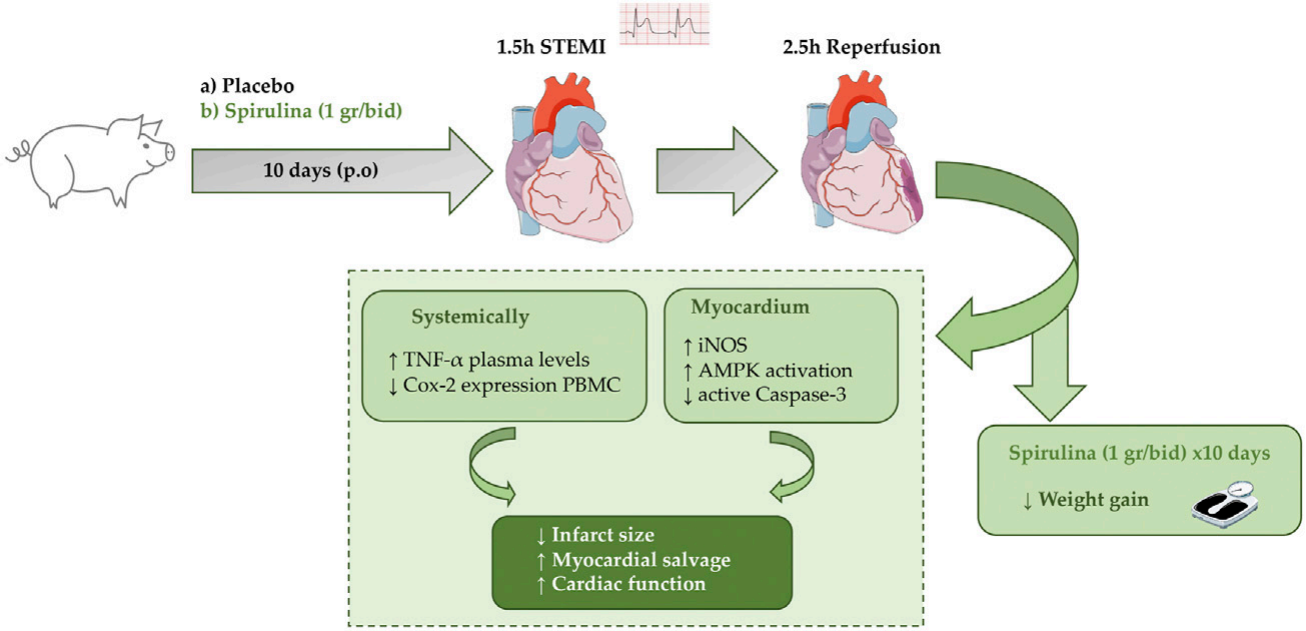
Study design and spirulina’s cardioprotective effects in a pig model of STEMI.

In conclusion, dietary spirulina supplementation for only 10 days has proven to elicit a cardioprotective effect in case of presentation of an ischemic event, through antioxidative, anti-inflammatory, and anti-apoptotic mechanisms that leads to a reduced size of infarction an improved cardiac function (Figure 7). Because of the absence of adverse effects, spirulina supplementation may represent a simple way to counteract the deleterious mechanism triggered during STEMI and may be a promising novel strategy to enhance myocardial salvage in the setting of MI.

## Data Availability

The original contributions presented in the study are included in the article/Supplementary Material, further inquiries can be directed to the corresponding author.

## References

[B1] Animal Ethics Infolink (2019). NSW Department of Primary Industries and Animal Research Review Panel. Available at: https://www.animalethics.org.au/three-rs .

[B2] Badimon L. Bugiardini R. Cenko E. Cubedo J. Dorobantu M. Duncker D. J. (2017). Position Paper of the European Society of Cardiology-Working Group of Coronary Pathophysiology and Microcirculation: Obesity and Heart Disease. Eur. Heart J. 38, 1951–1958. 10.1093/eurheartj/ehx181 28873951

[B3] Badimon L. Vilahur G. Padro T. (2010). Nutraceuticals and Atherosclerosis: Human Trials. Cardiovasc. Ther. 28, 202–215. 10.1111/j.1755-5922.2010.00189.x 20633023

[B4] Bolli R. (2001). Cardioprotective Function of Inducible Nitric Oxide Synthase and Role of Nitric Oxide in Myocardial Ischemia and Preconditioning: an Overview of a Decade of Research. J. Mol. Cell Cardiol 33, 1897–1918. 10.1006/jmcc.2001.1462 11708836

[B5] Carrizzo A. Izzo C. Forte M. Sommella E. Di Pietro P. Venturini E. (2020). A Novel Promising Frontier for Human Health: The Beneficial Effects of Nutraceuticals in Cardiovascular Diseases. Int. J. Mol. Sci. 21. 10.3390/ijms21228706 PMC7698807 33218062

[B6] Costa J. A. V. Freitas B. C. B. Rosa G. M. Moraes L. Morais M. G. Mitchell B. G. (2019). Operational and Economic Aspects of Spirulina-Based Biorefinery. Bioresour. Technol. 292, 121946. 10.1016/j.biortech.2019.121946 31422868

[B7] DiNicolantonio J. J. Bhat A. G. OKeefe J. (2020). Effects of Spirulina on Weight Loss and Blood Lipids: A Review. Open Heart 7, e001003. 10.1136/openhrt-2018-001003 32201580 PMC7061888

[B8] Finamore A. Palmery M. Bensehaila S. Peluso I. (2017). Antioxidant, Immunomodulating, and Microbial-Modulating Activities of the Sustainable and Ecofriendly Spirulina. Oxid Med. Cell Longev. 2017, 3247528. 10.1155/2017/3247528 28182098 PMC5274660

[B9] Fujimoto M. Tsuneyama K. Fujimoto T. Selmi C. Gershwin M. E. Shimada Y. (2012). Spirulina Improves Non-Alcoholic Steatohepatitis, Visceral Fat Macrophage Aggregation, and Serum Leptin in a Mouse Model of Metabolic Syndrome. Dig. Liver Dis. 44, 767–774. 10.1016/j.dld.2012.02.002 22444524

[B10] Grosshagauer S. Kraemer K. Somoza V. (2020). The True Value of Spirulina. J. Agric. Food Chem. 68, 4109–4115. 10.1021/acs.jafc.9b08251 32133854

[B11] Hassan H. A. El-Gharib N. E. (2015). Obesity and Clinical Riskiness Relationship: Therapeutic Management by Dietary Antioxidant Supplementation-Aa Review. Appl. Biochem. Biotechnol. 176, 647–669. 10.1007/s12010-015-1602-6 25864185

[B12] Hernández-Lepe M. A. Wall-Medrano A. López-Díaz J. A. Juárez-Oropeza M. A. Hernández-Torres R. P. Ramos-Jiménez A. (2019). Hypolipidemic Effect of Arthrospira (Spirulina) Maxima Supplementation and a Systematic Physical Exercise Program in Overweight and Obese Men: A Double-Blind, Randomized, and Crossover Controlled Trial. Mar. Drugs 17. 10.3390/md17050270 PMC6562443 31067674

[B13] Heusch G. Gersh B. J. (2020). Is Cardioprotection Salvageable? Circulation 141, 415–417. 10.1161/CIRCULATIONAHA.119.044176 32078426

[B14] Heusch G. Gersh B. J. (2017). The Pathophysiology of Acute Myocardial Infarction and Strategies of protection beyond Reperfusion: a Continual challenge. Eur. Heart J. 38, 774–784. 10.1093/eurheartj/ehw224 27354052

[B15] Heusch G. Libby P. Gersh B. Yellon D. Böhm M. Lopaschuk G. (2014). Cardiovascular Remodelling in Coronary Artery Disease and Heart Failure. Lancet 383, 1933–1943. 10.1016/S0140-6736(14)60107-0 24831770 PMC4330973

[B16] Huang H. Liao D. Pu R. Cui Y. (2018). Quantifying the Effects of Spirulina Supplementation on Plasma Lipid and Glucose Concentrations, Body Weight, and Blood Pressure. Diabetes Metab. Syndr. Obes. 11, 729–742. 10.2147/DMSO.S185672 30532573 PMC6241722

[B17] Jong W. M. Leemans J. C. Weber N. C. Juffermans N. P. Schultz M. J. Hollmann M. W. (2014). Nlrp3 Plays No Role in Acute Cardiac Infarction Due to Low Cardiac Expression. Int. J. Cardiol. 177, 41–43. 10.1016/j.ijcard.2014.09.148 25499334

[B18] Kanamori H. Takemura G. Goto K. Maruyama R. Tsujimoto A. Ogino A. (2011). The Role of Autophagy Emerging in Postinfarction Cardiac Remodelling. Cardiovasc. Res. 91, 330–339. 10.1093/cvr/cvr073 21406597

[B19] Karkos P. D. Leong S. C. Karkos C. D. Sivaji N. Assimakopoulos D. A. (2011). Spirulina in Clinical Practice: Evidence-Based Human Applications. Evid. Based Compl. Alternat Med. 2011, 531053. 10.1093/ecam/nen058 PMC3136577 18955364

[B20] Khan M. Shobha J. C. Mohan I. K. Naidu M. U. Sundaram C. Singh S. (2005). Protective Effect of Spirulina against Doxorubicin-Induced Cardiotoxicity. Phytother Res. 19, 1030–1037. 10.1002/ptr.1783 16372368

[B21] Khan M. Varadharaj S. Ganesan L. P. Shobha J. C. Naidu M. U. Parinandi N. L. (2006). C-phycocyanin Protects Against Ischemia-Reperfusion Injury of Heart through Involvement of P38 MAPK and ERK Signaling. Am. J. Physiol. Heart Circ. Physiol. 290, H2136–H2145. 10.1152/ajpheart.01072.2005 16373583

[B22] Khan M. Varadharaj S. Shobha J. C. Naidu M. U. Parinandi N. L. Kutala V. K. (2006). C-phycocyanin Ameliorates Doxorubicin-Induced Oxidative Stress and Apoptosis in Adult Rat Cardiomyocytes. J. Cardiovasc. Pharmacol. 47, 9–20. 10.1097/01.fjc.0000191520.48404.27 16424780

[B23] Lecour S. Smith R. M. Woodward B. Opie L. H. Rochette L. Sack M. N. (2002). Identification of a Novel Role for Sphingolipid Signaling in TNF Alpha and Ischemic Preconditioning Mediated Cardioprotection. J. Mol. Cell Cardiol 34, 509–518. 10.1006/jmcc.2002.1533 12056855

[B24] Lecour S. Suleman N. Deuchar G. A. Somers S. Lacerda L. Huisamen B. (2005). Pharmacological Preconditioning with Tumor Necrosis Factor-Alpha Activates Signal Transducer and Activator of Transcription-3 at Reperfusion without Involving Classic Prosurvival Kinases (Akt and Extracellular Signal-Regulated Kinase). Circulation 112, 3911–3918. 10.1161/CIRCULATIONAHA.105.581058 16344382

[B25] Li Q. Guo Y. Ou Q. Wu W. J. Chen N. Zhu X. (2011). Gene Transfer as a Strategy to Achieve Permanent Cardioprotection II: rAAV-Mediated Gene Therapy with Heme Oxygenase-1 Limits Infarct Size 1 Year Later without Adverse Functional Consequences. Basic Res. Cardiol. 106, 1367–1377. 10.1007/s00395-011-0208-6 21785893 PMC3640464

[B26] Li Q. Guo Y. Tan W. Stein A. B. Dawn B. Wu W. J. (2006). Gene Therapy with iNOS Provides Long-Term protection against Myocardial Infarction without Adverse Functional Consequences. Am. J. Physiol. Heart Circ. Physiol. 290, H584–H589. 10.1152/ajpheart.00855.2005 16172153 PMC3648984

[B27] Li T. T. Huang Z. R. Jia R. B. Lv X. C. Zhao C. Liu B. (2021). Spirulina Platensis Polysaccharides Attenuate Lipid and Carbohydrate Metabolism Disorder in High-Sucrose and High-Fat Diet-Fed Rats in Association with Intestinal Microbiota. Food Res. Int. 147, 110530. 10.1016/j.foodres.2021.110530 34399508

[B28] Liang Y. Huang X. Zhang Z. Deng K. An S. Gao X. (2020). Spirulina Supplementation Improves Lipid Metabolism and Autophagic Activities in the Liver and Muscle of Hu Lambs Fed a High-Energy Diet. Arch. Anim. Nutr. 74, 476–495. 10.1080/1745039X.2020.1820806 33059482

[B29] Madonna R. Cadeddu C. Deidda M. Giricz Z. Madeddu C. Mele D. (2015). Cardioprotection by Gene Therapy: A Review Paper on Behalf of the Working Group on Drug Cardiotoxicity and Cardioprotection of the Italian Society of Cardiology. Int. J. Cardiol. 191, 203–210. 10.1016/j.ijcard.2015.04.232 25974196

[B30] Mazokopakis E. E. Papadomanolaki M. G. Fousteris A. A. Kotsiris D. A. Lampadakis I. M. Ganotakis E. S. (2014). The Hepatoprotective and Hypolipidemic Effects of Spirulina (Arthrospira Platensis) Supplementation in a Cretan Population with Non-alcoholic Fatty Liver Disease: a Prospective Pilot Study. Ann. Gastroenterol. 27 (4), 387–394. 25331487 PMC4188938

[B31] Mazokopakis E. E. Starakis I. K. Papadomanolaki M. G. Mavroeidi N. G. Ganotakis E. S. (2014). The Hypolipidaemic Effects of Spirulina (Arthrospira Platensis) Supplementation in a Cretan Population: A Prospective Study. J. Sci. Food Agric. 94, 432–437. 10.1002/jsfa.6261 23754631

[B32] Mendieta G. Ben-Aicha S. Casani L. Badimon L. Sabaté M. Vilahur G. (2019). Intravenous Statin Administration during Ischemia Exerts Cardioprotective Effects. J. Am. Coll. Cardiol. 74, 475–477. 10.1016/j.jacc.2019.05.020 31319923

[B33] Mendieta G. Ben-Aicha S. Casani L. Badimon L. Sabate M. Vilahur G. (2019). Molecular Pathways Involved in the Cardioprotective Effects of Intravenous Statin Administration During Ischemia. Basic Res. Cardiol. 115, 2. 10.1007/s00395-019-0760-z 31781960

[B34] Mendieta G. Ben-Aicha S. Gutiérrez M. Casani L. Aržanauskaitė M. Carreras F. (2020). Intravenous Statin Administration during Myocardial Infarction Compared with Oral Post-Infarct Administration. J. Am. Coll. Cardiol. 75, 1386–1402. 10.1016/j.jacc.2020.01.042 32216907

[B35] Moradi S. Ziaei R. Foshati S. Mohammadi H. Nachvak S. M. Rouhani M. H. (2019). Effects of Spirulina Supplementation on Obesity: A Systematic Review and Meta-Analysis of Randomized Clinical Trials. Complement. Ther. Med. 47, 102211. 10.1016/j.ctim.2019.102211 31780031

[B36] Moran A. E. Forouzanfar M. H. Roth G. A. Mensah G. A. Ezzati M. Murray C. J. (2014). Temporal Trends in Ischemic Heart Disease Mortality in 21 World Regions, 1980 to 2010: the Global Burden of Disease 2010 Study. Circulation 129, 1483–1492. 10.1161/CIRCULATIONAHA.113.004042 24573352 PMC4181359

[B37] Nakaya Y. H. N. Goto Y. (1988). Cholesterol Lowering Effect of Spirulina. Nutr. Rep. Int.

[B38] Park H. J. Lee H. S. (2016). The Influence of Obesity on the Effects of Spirulina Supplementation in the Human Metabolic Response of Korean Elderly. Nutr. Res. Pract. 10, 418–423. 10.4162/nrp.2016.10.4.418 27478549 PMC4958645

[B39] Park H. J. Lee Y. J. Ryu H. K. Kim M. H. Chung H. W. Kim W. Y. (2008). A Randomized Double-Blind, Placebo-Controlled Study to Establish the Effects of Spirulina in Elderly Koreans. Ann. Nutr. Metab. 52, 322–328. 10.1159/000151486 18714150

[B40] Percie du Sert N. Ahluwalia A. Alam S. Avey M. T. Baker M. Browne W. J. (2020). Reporting Animal Research: Explanation and Elaboration for the ARRIVE Guidelines 2.0. PLoS Biol. 18, e3000411. 10.1371/journal.pbio.3000411 32663221 PMC7360025

[B41] Ren Z. Xie Z. Cao D. Gong M. Yang L. Zhou Z. (2018). C-phycocyanin Inhibits Hepatic Gluconeogenesis and Increases Glycogen Synthesis via Activating Akt and AMPK in Insulin Resistance Hepatocytes. Food Funct. 9, 2829–2839. 10.1039/c8fo00257f 29693104

[B42] Schulz R. Heusch G. (2009). Tumor Necrosis Factor-Alpha and its Receptors 1 and 2: Yin and Yang in Myocardial Infarction? Circulation 119, 1355–1357. 10.1161/CIRCULATIONAHA.108.846105 19255338

[B43] Serban M. C. Sahebkar A. Dragan S. Stoichescu-Hogea G. Ursoniu S. Andrica F. (2016). A Systematic Review and Meta-Analysis of the Impact of Spirulina Supplementation on Plasma Lipid Concentrations. Clin. Nutr. 35, 842–851. 10.1016/j.clnu.2015.09.007 26433766

[B44] Stone G. W. Selker H. P. Thiele H. Patel M. R. Udelson J. E. Ohman E. M. (2016). Relationship between Infarct Size and Outcomes Following Primary PCI: Patient-Level Analysis from 10 Randomized Trials. J. Am. Coll. Cardiol. 67, 1674–1683. 10.1016/j.jacc.2016.01.069 27056772

[B45] Vilahur G. Casaní L. Peña E. Crespo J. Juan-Babot O. Ben-Aicha S. (2018). Silybum marianum Provides Cardioprotection and Limits Adverse Remodeling post-myocardial Infarction by Mitigating Oxidative Stress and Reactive Fibrosis. Int. J. Cardiol. 270, 28–35. 10.1016/j.ijcard.2018.06.030 29936043

[B46] Vilahur G. Casaní L. Peña E. Duran X. Juan-Babot O. Badimon L. (2009). Induction of RISK by HMG-CoA Reductase Inhibition Affords Cardioprotection after Myocardial Infarction. Atherosclerosis 206, 95–101. 10.1016/j.atherosclerosis.2009.02.009 19419716

[B47] Vilahur G. Casani L. Peña E. Juan-Babot O. Mendieta G. Crespo J. (2014). HMG-CoA Reductase Inhibition Prior Reperfusion Improves Reparative Fibrosis post-myocardial Infarction in a Preclinical Experimental Model. Int. J. Cardiol. 175, 528–538. 10.1016/j.ijcard.2014.06.040 25023790

[B48] Vilahur G. Gutiérrez M. Casani L. Varela L. Capdevila A. Pons-Lladó G. (2016). Protective Effects of Ticagrelor on Myocardial Injury after Infarction. Circulation 134, 1708–1719. 10.1161/CIRCULATIONAHA.116.024014 27789556

[B49] Vilahur G. Badimon J. J. Bugiardini R. Badimon L. (2014). Perspectives: The burden of Cardiovascular Risk Factors and Coronary Heart Disease in Europe and Worldwide. Eur. Heart J. Suppl. 16, A7–A11. 10.1093/eurheartj/sut003

[B50] Vo T. S. Kim S. K. (2013). Down-Regulation of Histamine-Induced Endothelial Cell Activation as Potential Anti-atherosclerotic Activity of Peptides from Spirulina Maxima. Eur. J. Pharm. Sci. 50, 198–207. 10.1016/j.ejps.2013.07.001 23856417

[B51] Xi L. Jarrett N. C. Hess M. L. Kukreja R. C. (1999). Essential Role of Inducible Nitric Oxide Synthase in Monophosphoryl Lipid A-Induced Late Cardioprotection: Evidence from Pharmacological Inhibition and Gene Knockout Mice. Circulation 99, 2157–2163. 10.1161/01.cir.99.16.2157 10217657

[B52] Yordanova G. Popova T. Givko N. Nedeva R. Marchev Y. Kistanova E. (2015). Lipid Profile of M. Longissimus Dorsi in Response to Dietary Spirulina Platensis Supplementation in Pigs. Bulgarian J. Agric. Sci. 21, 1054–1059.

[B53] Yousefi R. Saidpour A. Mottaghi A. (2019). The Effects of Spirulina Supplementation on Metabolic Syndrome Components, its Liver Manifestation and Related Inflammatory Markers: A Systematic Review. Complement. Ther. Med. 42, 137–144. 10.1016/j.ctim.2018.11.013 30670232

[B54] Yu X. Ge L. Niu L. Lian X. Ma H. Pang L. (2018). The Dual Role of Inducible Nitric Oxide Synthase in Myocardial Ischemia/Reperfusion Injury: Friend or Foe? Oxid Med. Cell Longev. 2018, 8364848. 10.1155/2018/8364848 30510628 PMC6230379

[B55] Zhao B. Cui Y. Fan X. Qi P. Liu C. Zhou X. (2019). Anti-Obesity Effects of Spirulina Platensis Protein Hydrolysate by Modulating Brain-Liver axis in High-Fat Diet Fed Mice. PLoS One 14, e0218543. 10.1371/journal.pone.0218543 31220177 PMC6586325

